# Applying feminist principles to social work teaching: Pandemic times and beyond

**DOI:** 10.1177/1473325020973305

**Published:** 2021-03

**Authors:** Jessica Hutchison

**Affiliations:** Faculty of Social Work, Wilfrid Laurier University, Kitchener, Canada

**Keywords:** Pedagogy, feminism, social work education, social justice

## Abstract

It took a global pandemic for me to recognize how my social work teaching was an act of feminist praxis. I have long identified as a feminist and regularly engage efforts to advance equity for women, primarily centered on the abolition of prisons which disproportionately incarcerate Indigenous and Black women in Canada. Surprisingly, I have never considered how my feminism shows up in my teaching. The following reflexive essay explores the ways in which the feminist principles of centring emotions, rejecting patriarchal hierarchy, and challenging white feminism were embedded into the development and delivery of a graduate level social work research course that was rapidly adapted to being taught online during a global public health crisis. It ends with a call to action for social work educators to incorporate feminist principles into their pedagogies, not only in times of crisis, but as standard practice.

It took a global pandemic for me to recognize how my social work teaching was an act of feminist praxis. I have long identified as a feminist and regularly engage in efforts to advance equity for women, primarily centered on the abolition of prisons which disproportionately incarcerate Indigenous and Black women in Canada (Maynard, 2017). Surprisingly, I have never considered how my feminism shows up in my teaching. I am a white doctoral student in a Canadian social work program and have taught numerous post-secondary courses in social work and criminology. I teach from a critical perspective and centre care and compassion in the classroom, but I have never explicitly connected this to being a feminist. It is just how I teach. However, when reflecting on how I approached my social work teaching during the pandemic, I have discovered my pedagogy is decidedly and unapologetically feminist. The following reflexive essay explores the ways in which the feminist principles of centring emotions, rejecting patriarchal hierarchy, and challenging white feminism were embedded into the development and delivery of a graduate level social work research course that was rapidly adapted to being taught online during a global public health crisis. It ends with a call to action for social work educators to incorporate feminist principles into their pedagogies, not only in times of crisis, but as standard practice.

## Setting the stage

When I signed the contract to teach a research course in a Canadian Master of Social Work program, the COVID-19 pandemic was nowhere on the global radar. I was intentionally planning and designing a course that involved significant experiential components, and activities and assignments had been specifically designed for in-person delivery. However, in mid-March, the university ceased all in-person classes, and shifted them online, including the course I was slated to begin teaching four weeks later.

Despite personally navigating the unchartered territory of being confined to my house due to a deadly virus, at the forefront of my mind was, “How on earth am I going to teach research to social work students online?” Social work students do not typically identify as being skilled in research (Unrau and Grinnell, 2005) and the students in my class were no different. This is not surprising given universities are incubators for cultivating self-doubt in students’ abilities to think, act, and know (hooks, 2003), including in faculties of social work. One student’s comment seemed to reflect the feelings of the class, “Research has never been my strong suit. Taking an online research course worries me in terms of being able to grasp all the concepts and material.”

As I quickly redesigned the course to be delivered remotely, at the centre of my decision-making was consideration for how this shift to online learning will impact those who are already most disadvantaged. The class was comprised of mostly women (28 of 29 students), who I knew would bear the brunt of the burden of the pandemic due to patriarchy, capitalism, and white supremacy. They would be disproportionately impacted by increased child rearing demands, care for family and loved ones, and decreased financial security, and racialized women would feel the effects of these inequities more profoundly (Canadian Human Rights Commission, 2020).

I could have used the pandemic as a reason to engage in what Boler (2015: 1494) refers to as “drive-by difference”, which superficially acknowledges identity differences but does not meaningfully attend to the power inequities and emotions present in an online learning environment. However, knowing that these existing inequities and the stress that accompanies them would be exacerbated by the pandemic, signalled to the need to apply an equity lens to the development and delivery of the course.

## Feminist principles in teaching

Prior to the beginning of the course, I surveyed the students to get a sense of how they were feeling about the class and whether there was anything about their specific situation that was being impacted by the pandemic. The results revealed that students’ stress levels were high and that many students were now juggling full-time childcare, caring for ill family members, and trying to navigate working full or part-time while in graduate school full time. Below I describe some of the pedagogical decisions I made in response to students’ concerns and experiences in order to create the most just and equitable learning environment possible.

### Centring emotions

Feminists have often been critiqued for being too soft or emotional, particularly when it comes to employing emotion in the classroom (hooks, 2003). However, as Boler (2015: 1490) suggests, the centring of emotions in the classroom can, “Cultivate social justice within the politicized and hegemonic contexts of education and social justice”. Therefore, I used the power I held as the course instructor (Fisher, 2001) to attempt to address the exacerbated inequities that would result from the pandemic and made the conscious decision to teach from a foundation of compassion and love (hooks, 2003). This decision was a political one, rooted in social justice and equity. I made intentional pedagogical decisions, which I now recognize as being rooted in “feminist politics of emotions,” that challenge patriarchal epistemologies and traditional academic approaches by centring emotions and reframing them as normal responses to abnormal circumstances, rather than signs of pathology and weakness (Boler, 2015: 1492). If there was ever a time to acknowledge that emotions are a normal response to abnormal circumstances, a global health pandemic is it! Further, by understanding and treating emotions as a source of knowledge in the classroom, I was attempting to embody the ways in which emotions might also need to be considered as forms of knowledge in research. I was additionally signalling to students that attending to specificities of emotions at the micro-level (i.e., in the classroom) is a powerful act of solidarity and social justice, which can enable change at the macro-level (i.e., society).

I attempted to reduce the students’ worries by developing a course that centred care and love, and reduced as many structural barriers as possible, to lay the groundwork for the most optimal learning environment (hooks, 2003). I wrote this intention into the course syllabus:

*Due to the COVID-19 pandemic, the original in-person delivery of the course is not possible. I fully recognize this situation brings with it unforeseen stresses and responsibilities and I have redesigned the course to ensure flexibility and accommodation … Above all, I will be teaching from a place of compassion and understanding, while encouraging you to engage with the course content and each other.*

I recognized that while the purpose of the course was to help students develop their theoretical and practical understanding of social work research, my primary goal was to ensure the students were emotionally and cognitively cared for. I did not proceed with ‘business as usual’; instead, I attempted to model for the class that nothing about this time was ‘usual’ and that as training social workers, part of their future work will be to push back against these kinds of pressures.

While academia tends to erase caring as an intellectual concern (Fisher, 2001), caring has been shown to positively impact students’ academic engagement, enjoyment of coursework, and mastering of difficult material (Meyers, 2009). Indeed, in the end-of-course evaluation a student commented, “Thank you for being so attentive and empathetic to the fears and worries of your students who were a bit apprehensive about the material in this course. I gained a lot of knowledge from the course material.” Not only did I demonstrate my care and concern for students through my discourse and tone, I also enacted structural changes that reduced barriers to engagement, as described next.

### Rejecting patriarchal hierarchy

Given my rejection of the patriarchal, hierarchal model of teaching that privileges control and authority, I worked to centre choice, autonomy, and learning. I chose to teach the course asynchronously so as not to disadvantage those who could not attend synchronous classes due to conflicting demands of parenting, caregiving, and employment. Lessons were released one week at a time, with a break every three weeks to allow for students to catch up on work they had fallen behind on and to allow them to integrate what they were learning. This allowed students to work through the material when it was possible for their lives, and rejected the idea that increased quantity of content means students will learn more. Surprisingly, one of the most appreciated aspects of the online learning space was the inclusion of a ‘task list’ at the beginning of each lesson. Many students reported that they printed this list and checked each item off as they completed it, which gave them a sense of accomplishment and control in chaotic times. Optional drop-in sessions via Zoom with me were offered each week to provide students with a sense of community in these socially isolating times and to give them a chance for individual or communal support, feedback, and content integration.

Similarly, I eliminated the use of hard deadlines. Suggested due dates were provided in recognition that some students work best with a bit of structure; however, they were never enforced. Students had the power to decide when to complete and submit course assignments. When students felt they needed to be held accountable for getting their work done, we engaged in collaborative dialogue around what accountability would look like. Due to my abolitionist values, I did not want to enact punitive measures; however, some students asked to have late penalties applied, which I complied with when asked to do so. While the floating due dates resulted in more work for me, feedback from students throughout the semester was that this approach was “a saving grace.”

Through rejecting patriarchal hierarchy, I demonstrated a more equitable and just approach to teaching is possible. However, pedagogy needs to attend to other power structures in addition to gender.

### Challenging white feminism

White feminism’s focus on the struggles of middle class white women, to the exclusion of poor and racialized women, has done significant harms, and is merely concerned with elevating straight, white, middle class women to the status of white, middle class men. Further, social work education and practice is dominated by whiteness (Badwall, 2015), both in terms of numbers – which was the case for my class where myself and the majority of the students were white – and in its normative practices. Given my rejection of white feminism and commitment to an intersectional feminist perspective, a primary goal of the course was for students to develop critical thinking and research skills regarding race, gender, ability, class, and other power structures that influence how people experience the world. I intentionally incorporated activities and discussions around contemporary socio-political issues, such as the pandemic, anti-Black racism and state violence, and the resistance movement of Black Lives Matter.

For example, in the first lesson, I framed COVID-19 as a social justice issue and students listened to a podcast that discussed the question of whether the coronavirus was an equalizer or magnifier (Walters and Pate, 2020). Students were asked to reflect on the question: “Are there communities/groups of people who are disproportionately impacted by COVID-19, and if so, why might this be?” I view such activities as crucial components of social work education, as they challenge the dominant white, middle class worldview of academia and social work. Similarly, when selecting a data set for a qualitative analysis assignment, I intentionally centered interviews with racialized participants. One of the racialized students in the class thanked me for doing this, which suggests that research centering racialized perspectives was not a common experience in her social work education.

Furthermore, when George Floyd was murdered by a police officer in Minneapolis exactly halfway through our course, the groundwork had already been laid for attending to emotions within the classroom, critically engaging with issues of race and racism, and connecting current social and political events to research. Many students indicated that they had been deeply emotionally impacted by this racist killing, and I was too. To bear witness to the modern-day lynching of a Black man by a white man casually kneeling on his neck for 8 minutes and 46 seconds until he died, shook me to the core. I had a visceral reaction that felt like molten lava coursing through my veins. I was enraged. I could not concentrate on lesson planning or grading assignments, which, up until this point, I was on top of. I considered cancelling the lesson that was scheduled for the Wednesday after his murder, because to be honest, who cares about research when Black people were being lynched in the street?

However, reminding myself that the purpose of this course was for students to develop research skills to contribute to social justice and transformational change, and that my feelings of rage as a white woman did not absolve me from needing to be accountable in my spheres of influence, in this case the classroom, helped me to return to my pedagogical principles. My responsibility as the instructor was to both make room for these emotions to be expressed, and to help students see the connections between this horrifying injustice and social work research that has the potential for being complicit in maintaining the status quo or be utilized as a tool for dismantling harmful systems. The lesson that week was coincidentally about the transformative potential of participatory research methods, which centres the voices of the people most impacted by the issue being studied, with the specific goal of effecting social change (Leavy, 2017). The parallels between participatory research and the global Black Lives Matter movement provided a solid foundation from which to help students develop an analysis of how research can be liberatory. For example, the reading that week was a participatory action research project undertaken with survivors of sex trafficking in Nepal (Dhungel et al., 2019). In order to apply current events to the liberatory potential of participatory research outlined in the article, students were asked to do the following:

*Reflecting on the statement, “We claim that solidarity and unity are powerful, and they helped amplify our collective voices to advocate for social change and our emancipation” (*Dhungel et al., 2019*: 53), what comes to mind with respect to what is happening in response to anti-Black racism in Canada and the U.S.? How can participatory research be used to elicit transformational change?*

By linking what students were reading to the global movement for freedom from anti-Black racism and state violence, students learned that social work research is not just an academic exercise that remains in the ivory tower. Rather, it has powerful liberatory and transformational potential when done in true solidarity with those most affected by the issues.

## Concluding thoughts

While it took a global health crisis for me to identify my pedagogy as feminist, it is clear the approach created a more socially just learning environment. I will be incorporating feminist principles into my future teaching, and I suggest all social work educators do, given social work’s commitment to social justice and transformational change.
